# Neurophysiological signatures of Alzheimer’s disease and frontotemporal lobar degeneration: pathology versus phenotype

**DOI:** 10.1093/brain/awy180

**Published:** 2018-07-09

**Authors:** Saber Sami, Nitin Williams, Laura E Hughes, Thomas E Cope, Timothy Rittman, Ian T S Coyle-Gilchrist, Richard N Henson, James B Rowe

**Affiliations:** 1 Department of Clinical Neurosciences, University of Cambridge, UK; 2 Neuroscience Center, University of Helsinki, Finland; 3 Medical Research Council Cognition and Brain Sciences Unit, Cambridge, UK

**Keywords:** effective connectivity, magnetoencephalography, Alzheimer’s disease, frontotemporal dementia, dementia

## Abstract

The disruption of brain networks is characteristic of neurodegenerative dementias. However, it is controversial whether changes in connectivity reflect only the functional anatomy of disease, with selective vulnerability of brain networks, or the specific neurophysiological consequences of different neuropathologies within brain networks. We proposed that the oscillatory dynamics of cortical circuits reflect the tuning of local neural interactions, such that different pathologies are selective in their impact on the frequency spectrum of oscillations, whereas clinical syndromes reflect the anatomical distribution of pathology and physiological change. To test this hypothesis, we used magnetoencephalography from five patient groups, representing dissociated pathological subtypes and distributions across frontal, parietal and temporal lobes: amnestic Alzheimer’s disease, posterior cortical atrophy, and three syndromes associated with frontotemporal lobar degeneration. We measured effective connectivity with graph theory-based measures of local efficiency, using partial directed coherence between sensors. As expected, each disease caused large-scale changes of neurophysiological brain networks, with reductions in local efficiency compared to controls. Critically however, the frequency range of altered connectivity was consistent across clinical syndromes that shared a likely underlying pathology, whilst the localization of changes differed between clinical syndromes. Multivariate pattern analysis of the frequency-specific topographies of local efficiency separated the disorders from each other and from controls (accuracy 62% to 100%, according to the groups’ differences in likely pathology and clinical syndrome). The data indicate that magnetoencephalography has the potential to reveal specific changes in neurophysiology resulting from neurodegenerative disease. Our findings confirm that while clinical syndromes have characteristic anatomical patterns of abnormal connectivity that may be identified with other methods like structural brain imaging, the different mechanisms of neurodegeneration also cause characteristic spectral signatures of physiological coupling that are not accessible with structural imaging nor confounded by the neurovascular signalling of functional MRI. We suggest that these spectral characteristics of altered connectivity are the result of differential disruption of neuronal microstructure and synaptic physiology by Alzheimer’s disease versus frontotemporal lobar degeneration.

## Teaser

Sami *et al.* identify characteristic neurophysiological signatures of five neurodegenerative diseases, including two variants of Alzheimer’s disease and three forms of frontotemporal lobar degeneration. Disorders that share a common underlying pathology have a similar spectral signature of altered connectivity, regardless of phenotype.

## Introduction

The impact of neurodegeneration can be understood in terms of its effect on the structure and function of brain networks. For example, there are structural anatomical fingerprints for Alzheimer’s disease and frontotemporal dementia, and disease-specific changes in their functional connectivity with ‘epicentres’ of disease (Seeley *et al.*, 2009; Zhou *et al.*, 2010; Crossley *et al.*, 2014). Moreover, the distribution of abnormal connectivity mirrors the anatomical and functional networks in health, suggesting selective vulnerability of brain networks to neuropathology (Pievani *et al.*, 2011).

The evidence for network-specific changes in major human dementia syndromes comes largely from functional MRI. However, MRI indirectly examines the physiological consequences of neuropathology. For example, in Alzheimer’s disease misfolding and aggregation of amyloid-β and microtubule-associated protein tau (MAPT), occur in a cascade that ultimately impacts on synaptic function and cell survival (Spires-Jones and Hyman, 2014). Functional MRI can detect the late consequences of this cascade on connectivity (Bullmore and Sporns, 2009; Fornito *et al.*, 2015), but it is limited by slow and indirect neurovascular signalling (Hillman, 2014; Tsvetanov *et al.*, 2015). In contrast, magnetoencephalography (MEG) and EEG offer a temporal resolution that can resolve changes in neural dynamics that are indistinguishable by functional MRI, and that are independent of effects of age or medication on the neurovascular response (de Haan *et al.*, 2012*a*; Hughes *et al.*, 2013; Tsvetanov *et al.*, 2015).

Clinical research applications of EEG have reported features that distinguish Alzheimer’s disease from controls (Triggiani *et al.*, 2017); predict the conversion from mild cognitive impairment to dementia (Poil *et al.*, 2013); and provide pre-symptomatic markers of autosomal dominant disease (Quiroz *et al.*, 2011). In contrast, clinical EEGs of frontotemporal dementia are often regarded as normal, although abnormalities at the group level have been shown (Chan *et al.*, 2004). Beyond clinical applications, the spectral and spatial resolution of MEG and EEG enables one to test key hypotheses of human neurodegeneration; identifying the reorganization of networks in dementia; and providing potential biomarkers for diagnosis, prognosis or drug response (Hughes and Rowe, 2013; Hughes *et al.*, 2013, 2015; Maestu *et al.*, 2015).

Here, we exploit the spatiotemporal precision of MEG to build on preclinical models of dementia, and determine the specificity of pathophysiological signatures of Alzheimer’s disease pathology versus frontotemporal lobar degeneration (FTLD). For example, transgenic rodent models of Alzheimer’s disease have indicated specific alterations in fast network dynamics, resulting in loss of gamma power (30+ Hz) in cortical and hippocampal local networks (Kurudenkandy *et al.*, 2014). Analogous changes in network dynamics can be identified in humans, noting that the distribution of disease can vary between early medial-temporal lobe changes in typical Alzheimer’s disease, versus an occipito-parietal focus in the posterior cortical atrophy (PCA) variant (Selkoe, 2002; Crutch *et al.*, 2012; Pena-Ortega *et al.*, 2012). The behavioural variant of frontotemporal dementia (bvFTD), on the other hand, is often associated with tauopathy in the absence of amyloid-β, for which electrophysiological recordings in rodent tauopathy models indicate power reductions in the lower frequency bands; alpha (8–13 Hz) and beta (14–30 Hz) (Koss *et al.*, 2016). FTLD also encompasses the non-fluent agrammatic variant of primary progressive aphasia (navPPA) and progressive supranuclear palsy (PSP, Richardson’s syndrome), which are both most commonly caused by primary tauopathy but which differ in the severity and location of atrophy (Ghosh *et al.*, 2012; Mandelli *et al.*, 2016).

Our overarching hypothesis was that different neuropathologies have characteristic physiological signatures, which reflect both the anatomical distribution of pathology and their impact on the oscillatory dynamics of cortical circuits. We predicted that typical Alzheimer’s disease and PCA would differ in the localization of their functional effects, but that the changes in oscillatory dynamics within affected regions would be similar. In contrast, we predicted that three subtypes of FTLD would have different spectral and spatial properties compared to Alzheimer’s disease, while their spectral properties may be similar to each other albeit in different spatial distributions and with distinct clinical phenotypes. The significance of MEG-based differentiation of these five syndromes is not primarily for utility as a diagnostic biomarker, in competition with other biomarkers. Rather, it lies in establishing their pathophysiological signatures in humans *in vivo*, extending the network paradigm of neurodegeneration to the spectral domain, and validating translational models of disease.

Our principle measure of network function was local efficiency, which indicates a network’s local information transfer and resilience (Bullmore and Sporns, 2009; Stam, 2014). This measure is therefore ideally suited to examine the effect of degenerative syndromes associated with regional variations in pathology. Note that locality here refers to topological locality, and not Euclidean locality or physical proximity. The reorganization of brain networks can also be measured in terms of global properties (e.g. small worldness), or the changes in the properties of hub regions that are critical for effective long-range integration (Crossley *et al.*, 2014). However, we focus on local efficiency as although neurodegeneration is diffuse, it is not uniformly distributed: both frontotemporal dementia and Alzheimer’s disease variants like PCA manifest clear regional specificity, in keeping with their nomenclature. Moreover, a potential advantage of the neurophysiological approach is greater sensitivity to network reorganization before extensive cell death leads to atrophy (Knight and Verkhratsky, 2010; Palop and Mucke, 2010; Hughes *et al.*, 2013).

## Materials and methods

### Participants

Patients were enrolled from tertiary clinics at Cambridge University Hospitals NHS Trust. Patients with Alzheimer’s disease included 13 with typical Alzheimer’s disease (McKhann *et al.*, 2011) and 11 with PCA (Crutch *et al.*, 2012). Patients with clinical syndromes associated with FTLD comprised 13 patients with bvFTD (Rascovsky *et al.*, 2011), all with abnormal structural MRI and evidence of progression; 15 with PSP (Litvan *et al.*, 1996) and 11 with navPPA (Gorno-Tempini *et al.*, 2011). The PSP cases meet the definition for probable or definite PSP-Richardson’s syndrome under the revised diagnostic criteria (Höglinger *et al.*, 2017). Fifteen healthy adult participants were recruited (11 males; age 59–85 years) with no history of neurological or psychiatric illness. The study was approved by the local Research Ethics Committee and written informed consent was obtained in accordance with the standards of the Declaration of Helsinki. Patients undertook the Mini-Mental State Examination (MMSE) and the revised Addenbrooke’s cognitive examination (ACE-R). The clinical and cognitive features of the patients are summarized in Table 1.

**Table 1:** Clinical and neuropsychological data for patient participants

**Group**	**Controls**	**tAD**	**PCA**	**BvFTD**	**NavPPA**	**PSP-RS**
*n*	15	13	11	13	11	15
Age	66.3 ± 5.9	71.3 ± 7.4	60.5 ± 4.5*	64.3 ± 6.9	72 ± 8.7	67.9 ± 6.5
MMSE (0–30)	29.4 ± 0.7	25.0 ± 3.2***	19.6 ± 6.0***	24.6 ± 3.8***	27.9 ± 2.2*	27.0 ± 2.8**
ACE-R (0–100)	96.5 ± 4.4	71.5 ± 8.2***	53.7 ± 21.3***	71.8 ± 14.8***	84.3 ± 11.6**	83.2 ± 7.9***
ACE-R memory (0–26)	25.2 ± 1.0	12.7 ± 3.6***	13.6 ± 7.8***	17.9 ± 5.5***	21.6 ± 6.9	22.0 ± 4.0**

Data are presented as mean ± SD.

ACE-R = revised Addenbrooke’s Cognitive Examination, total (0–100) and its memory subscale (0–26); MMSE = Mini-Mental State Examination; RS = Richardson’s syndrome; tAD = typical amnestic Alzheimer’s disease.

Differences between each patient group and controls: *P < 0.05; **P < 0.01; ***P < 0.001 uncorrected.

### Experimental design and data acquisition

**Figure 1:**
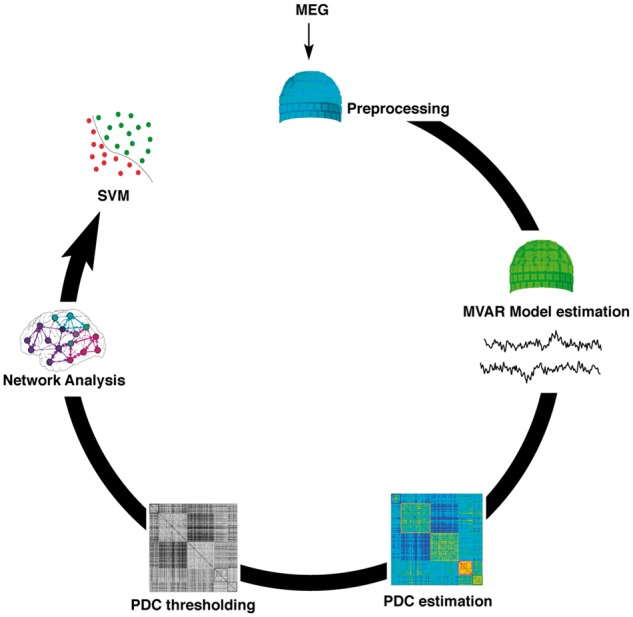
**Data analysis pipeline.** *Clockwise from top*: Preprocessing, to remove biological artefacts using MaxFilter and independent component analysis denoising; estimation of effective connectivity using MVAR; compiling the association matrix between sensors by PDC; applying a statistical threshold to create a binarized graph, represented by the connectivity matrix; graph network analysis to estimate local efficiency; and group classification using a support vector machine (SVM).

Figure 1 illustrates the flow of the processes involved in the data acquisition, preprocessing and analysis. All participants rested with their eyes closed while MEG was continuously recorded at 1 kHz sampling rate from 204 planar gradiometers using a Vectorview system (Elekta Neuromag) within a magnetically shielded room. The first 30–40 s of data were discarded to allow the participant to settle, resulting in 4 min of data per participant. Horizontal and vertical electrooculograms (EOG) were recorded and the participants’ head position was tracked with five head position indicator coils, localized in 3D together with ∼100 head points for anatomical registration using a 3D digitizer (Fastrak Polhemus, Inc.). The removal of environmental artefacts and head position alignment used the temporal extension of Signal Space Separation (tSSS) with Elekta-Neuromag MaxFilter v.2.2. Oculomotor artefacts were removed by independent component analysis, followed by projection out of the data of those independent components that correlated highly with either of the two EOG signals (typically 1–3 independent components per participant) (Gonzalez-Moreno *et al.*, 2014).

To examine the effect of neurodegeneration on network connectivity, we applied multivariate autoregressive modelling (MVAR) to the root mean square of the two planar gradiometers at each of the 102 locations around the head. One advantage of MVAR is that it ignores zero-lag correlations, which include those arising from volume conduction of a single brain source to multiple sensors. This reduces the need for source reconstruction of the MEG data; an inverse problem that cannot be solved without additional assumptions.

To reduce dimensionality and zero-lag co-linearity, and increase Gaussianity, we performed principal components analysis on the root mean square data and retained the first 60 principal components (which accounted for over 99% of the total variance). The MVAR was fitted to the lagged covariance matrices using the Vieira-Morf method. For MVAR modelling, an important step is to specify the model order, *p* (i.e. the number of past samples needed to predict the current sample). To find the optimal balance between model fit and complexity, we used the Bayesian information criterion, which resulted in an optimal model order of *p* = 8 for all groups. To test whether the data were stationary we used the stability index: data are stationary if and only if stability index < 0. This confirmed that the MVAR modelling did not significantly deviate from stationarity in any participant.

After projecting the fitted MVAR parameters back into sensor space, we estimated partial directed coherence (PDC) (Baccalá and Sameshima, 2001; Schelter *et al.*, 2006; Williams *et al.*, 2014) between all 102 sensor locations. PDC is a frequency-specific measure of connectivity that preserves the directionality of interactions, and has been shown to provide good reliability for group studies (Colclough *et al.*, 2016). PDC was estimated every 0.1 Hz from 1 to 80 Hz to create a 102 × 102 matrix for each of 800 frequencies, using the significance for which the *P*-values threshold was then binarized by taking the top 15% of significant connections for each frequency bin (i.e. a threshold of 85%, based on simulations below). The binary connectivity matrices were then used to estimate local efficiency for each node (location) and frequency.

### Simulations

To validate the ability of MVAR modelling to detect local network difference in functional segregation, we created 20 source-level networks with low mean local efficiency and 20 with high mean local efficiency. Each of the networks had six sources that differed in which six of the possible 30 connections existed. The sources corresponded to dipoles positioned on the cortical surface of a single-subject brain that was warped to MNI space. The dipoles were those closest to points in left and right prefrontal cortex (MNI coordinates of [±60 +10 +20]), left and right parietal cortex (MNI coordinates of [±50 − 70 +30]) and left and right ventral temporal cortex (MNI coordinates of [±30 − 70 0]). Multivariate time series of samples, with a sampling frequency of 1 kHz, were generated from an MVAR process with a zero-mean, unit variance independent Gaussian innovation for each source. These time series were then projected through a forward model that was based on a deformed sphere approximation to the individual’s inner skull surface, which had been co-registered with the MEG sensors from a real recording from the Elekta VectorView system. We added independent Gaussian noise to each of the simulated gradiometer time series, with a variable signal-to-noise ratio (SNR) based on the standard deviation (SD) of signal to noise, with SNR values of [0.1, 1, 3, 10, Inf] (where Inf means no added sensor noise). We then fitted the sensor-level data with an MVAR model, and generated PDC matrices to assess whether sensor-level networks preserved differences in mean local efficiency of the underlying source-level networks. These assessments were performed with PDC matrices binarized with thresholds of 85%, 95%, and 99%, respectively.

### Statistical analysis

To correct for multiple tests across sensors and frequencies in the MEG analysis, we projected the 102 sensor locations onto a 2D plane, and interpolated their local efficiency values onto a 64 × 64 grid using SPM8 (http://www.fil.ion.ucl.ac.uk/spm), resulting in a 3D scalp × frequency image (64 × 64 × 800), which was smoothed with a Gaussian kernel (8 mm × 8 mm × 8 Hz). Pairwise differences between groups were then assessed with Statistical non-Parametric Mapping (SnPM, http://warwick.ac.uk/snpm), which used 5000 permutations to generate pseudo-T distributions that are robust to small sample sizes and do not assume Gaussian error. A cluster-based extension was implemented to detect statistically significant clusters on t-maps (Hayasaka and Nichols, 2004). The cluster value was set to conform to the 95th percentile of the data-driven distribution, and significant clusters were set to *P < *0.05. For interpretation, the frequencies were subdivided into delta (<4 Hz), theta (4–7 Hz), alpha (8–13 Hz), beta (14–30 Hz), gamma (30–50 Hz), and high-gamma activity (>50 Hz) bands.

### Classification of participants

In the final analyses, we used multivariate pattern classification to identify the distinctions between two or more groups, enabling subject-specific group assignment based on the spatio-spectral characteristics of the networks’ local efficiency. Pattern recognition was implemented in MATLAB (R2012b; Mathworks, Natick, MA) using the Mania toolbox (https://bitbucket.org/grotegerd/mania), which incorporates the LIBSVM software library for the kernel-based support vector classification used (https://www.csie.ntu.edu.tw/∼cjlin/libsvm/). Support vector machines automatically calculate decision boundaries (hyperplanes) in a high-dimensional feature space based on training data with known outcome; new data are then placed into this space and outcome (prediction accuracy) determined according to its position relative to the hyperplane.

We used linear kernels for the support vector machine classification with parameters bounded between (0,1).

Before classification, we extracted low dimensional data features to improve performance, by using (i) a Z-statistic that was calculated over every voxel; and (ii) principal components analysis of the 3D frequency × scalp image of local efficiency. We applied leave-one-out cross-validation, iteratively dividing the data into separate training and testing sets with balanced groups and re-ran this iteration 10-fold to ensure stability. Finally, the classification performance from the support vector machine is described in terms of the area under the curve (AUC) of a receiver operating characteristic (ROC).

## Results

A summary of demographic and clinical measures for the patient groups is reported in Table 1. Across the five clinical groups and controls, there was a group-wise difference in age *P < *0.05. However, Tukey HSD tests and Holm correction for multiple comparisons, confirmed that only the PCA group were distinct in age, being younger (Table 1). All groups differed from controls in MMSE and ACE-R scores, as expected (Table 1).

**Figure 2:**
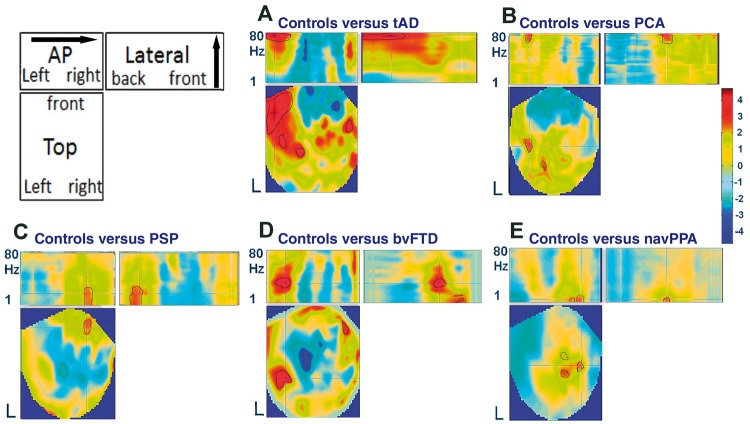
**3D scalp-frequency images of local efficiency.** The schematic (*top left*) indicates the three projections: the topography (Top) for each frequency (1–80 Hz), an anterior-posterior (AP) projection, separating frequency but collapsing over left–right axis, and a ‘lateral’ view, separating by frequency but collapsing over anterior-posterior axis. The five subplots (**A**–**E**) indicate the T-value for the difference in local efficiency for each patient group versus controls (colour bar on *right*). The cross-hair shows the peak T-statistic, while the black outline indicates regions surviving a cluster-corrected threshold of *P < *0.05.

Figure 2 shows sections through the 3D scalp frequency images of statistical differences between groups in local efficiency. Compared to healthy control subjects, patients with typical Alzheimer’s disease reduced local efficiency over temporal cortex (Fig. 2A). This effect was not equivalent across all frequencies, but was observed in the gamma range. The PCA variant of Alzheimer’s disease also caused a similar reduction in gamma band local efficiency, but in a different distribution that lay over more posterior regions (Fig. 2B).

A physiologically distinct signature was observed for the FTLD syndromes. For both PSP and bvFTD (Fig. 2C and D), local efficiency was reduced in lower frequencies, extending from delta through alpha to low gamma. These changes were evident over frontal cortex. While navPPA showed a similar reduction at low frequencies particularly in the delta/theta range, the distribution of the changes was different to that seen in bvFTD and PSP, focused on centro-parietal regions (Fig. 2E).

**Figure 3:**
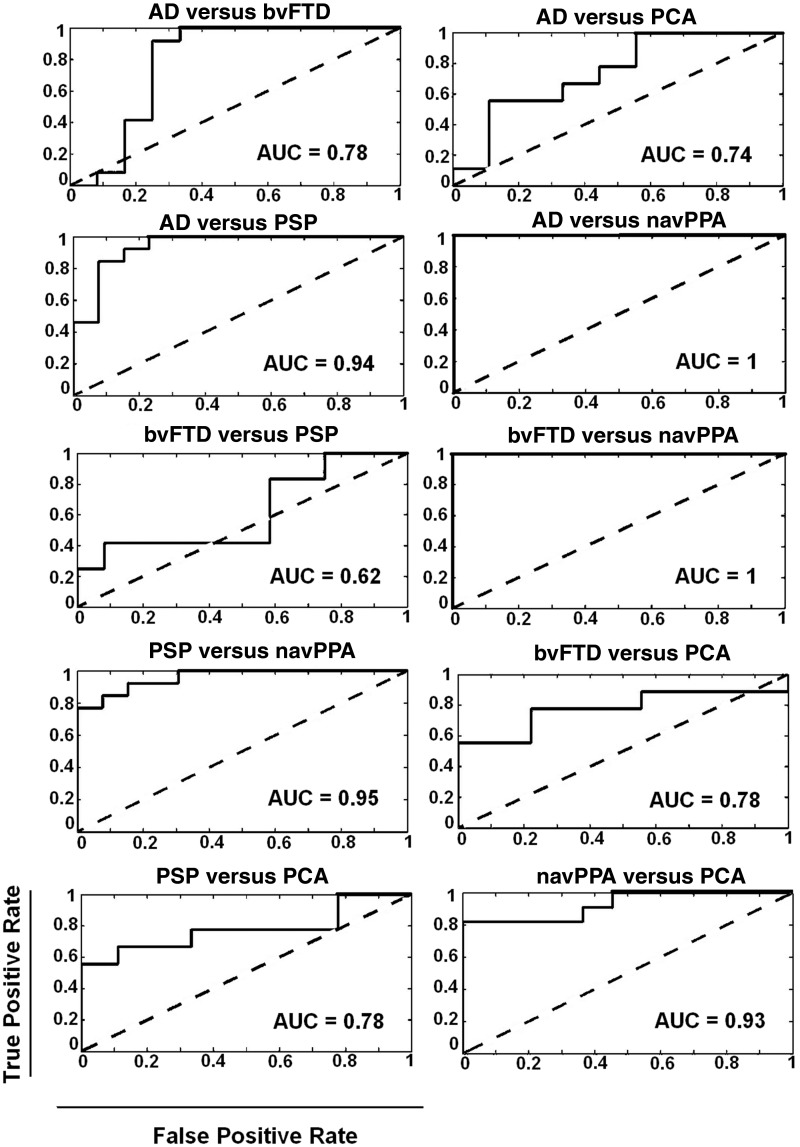
**ROC curves illustrate the performance of the support vector machine classifier following principle component feature extraction for binary classifications between each pair of patient groups.** The classification performance between patient groups is summarized by the area under the curve (AUC, *inset*). AD = Alzheimer disease.

Figure 3 shows ROCs for each binary classification of patient groups, based on the spatial and spectral distributions of local efficiency. Across all the comparisons of Alzheimer’s disease variants versus FTLD variants, classification performance (AUC) ranged from 0.78 to 1. AUC for distinguishing between the two Alzheimer’s disease variants typical Alzheimer’s disease and PCA was 0.74, while that for distinguishing the three frontotemporal dementia variants ranged from 0.62 (bvFTD versus PSP) to 1 (bvFTD versus navPPA).

Supplementary Fig. 1 shows ROCs for classification of each patient group versus controls, based on the spatial and spectral distributions of local efficiency. The difference was highest for bvFTD group followed by typical Alzheimer’s disease, with classification performance (AUC) at 0.96 and 0.85, respectively, and above-chance lower classification rates for PSP (0.76), navPPA (0.63) and PCA (0.60).

From the simulations, we confirmed that differences in mean local efficiency of the source-level networks were preserved in mean local efficiency of the corresponding sensor-level networks, at least for SNR levels of 3 and above when thresholding at 85% and 95% (Supplementary Table 1). Supplementary Fig. 2 illustrates this graphically on an example of a low mean local efficiency and a high mean local efficiency network.

### Data availability

Anonymized data will be shared on request from a qualified investigator for purposes of replicating procedures and results, and will be shared on request from a qualified investigator for other non-commercial research purposes within the limits of participants' consent.

## Discussion

We have identified distinctive neurophysiological signatures associated with five neurodegenerative disorders resulting from Alzheimer’s disease and FTLD. The signatures are characterized in terms of both the spatial and frequency profiles of local efficiency in brain networks (local in topological terms). The anatomical distribution of changes in local efficiency across the five syndromes was predicted by the functional anatomy of their principal cognitive deficits. However, the disorders were also distinguished by their spectral pattern of connectivity, according to the likely underlying neuropathology.

In recent years the majority of MEG-based dementia-related studies have focused on potential diagnostic or prognostic biomarker utility (Stam, 2014). We confirm that the spectral dynamics and topography of local efficiency enabled classification of patients using a simple machine-learning approach, with highest accuracy for classification between disorders. However, in the following discussion, we evaluate our results in relation to Alzheimer pathology, FTLD and the insights into pathogenic mechanisms from a network-based approach.

We focus on local network efficiency as one of the strongest indicators of underlying pathology in Alzheimer’s disease and frontotemporal dementia (Stam, 2014; Medaglia *et al.*, 2017). Other graph network metrics have been reported to be sensitive to the effects of neurodegeneration, including measures of global integration or organization. For example, previous MEG studies have shown the importance of network modularity and of hub connections in Alzheimer’s disease (de Haan *et al.*, 2012*b*; Stam, 2014), while a multi-layer frequency-band approach reveals the disruption of hubs in Alzheimer’s disease patients (Yu *et al.*, 2017). Recent simulations demonstrate the link between directed functional connectivity and hubs (Moon *et al.*, 2015; Meier *et al.*, 2017). This challenges the simple dichotomy between ‘local’ or ‘global’ integration, as hubs are themselves unevenly distributed yet influence global connectivity (Stam, 2014). Conversely, the changes in local efficiency we observed in Alzheimer’s disease are widespread across the brain. Distributed changes in local efficiency metrics may also contribute to part of the frequently reported change in global efficiency and hub connectivity in multiple neurological disorders (Crossley *et al.*, 2014). For example, closeness centrality is a characteristic of hubs that is directly proportional to their local efficiency (Sporns *et al.*, 2007). Our results highlight the distributed disruption of characteristic spectral signatures of physiological coupling in neurodegenerative disorders. Additionally, the local efficiency metric signifies tolerance in a network to a node’s removal i.e. a subgraph or a local network’s vulnerability is related to the reduction of the local efficiency of its contributing regions or nodes (Latora and Marchiori, 2001; Medaglia *et al.*, 2017). This is of special interest in view of the impact of neuropathological burden as measured by PET on connectivity (Cope *et al.*, 2018).

While network degeneration can be characterized at the microscopic level, or at the level of global brain function, we measured network dynamics at an intermediate scale, to reflect the regional variations in pathology in each of the five disorders. In the context of brain network dynamics, we confirmed the hypothesis that disorders that share a common underlying pathology have a similar spectral signature of altered connectivity, regardless of phenotype. This neurophysiological finding is distinct from the previously established relationships between the structural connectome, pathology and syndrome (Raj *et al.*, 2012; Zhou *et al.*, 2012).

### Alzheimer’s disease

Previous work in Alzheimer’s disease models and patients has demonstrated reductions in relative spectral power in high beta-gamma bands (Poza *et al.*, 2007), despite methodological differences between electrophysiological studies (Dauwels *et al.*, 2010). For example, in a network analysis using EEG, Petersen *et al.* (2001) found small-worldness of networks in the gamma band correlated with cognitive performance in prodromal Alzheimer’s disease. Preclinical models have investigated the aetiology of these changes, including the analogous loss of gamma power and theta-gamma coupling in cortical and hippocampal local networks in transgenic mouse models (Kurudenkandy *et al.*, 2014). For example, amyloid-β suppresses power in the beta-gamma frequency range from entorhinal cortex and induces desynchronization of pyramidal cells with a shift of the excitatory-inhibitory equilibrium (Pena-Ortega *et al.*, 2012; Kurudenkandy *et al.*, 2014). Neurodegenerative disease processes not only reduce synaptic density on pyramidal cells, and their local interactions with inhibitory interneurons, but also reduce neurotransmitters such as GABA (Selkoe, 2002; Huey *et al.*, 2006). Given the key role of GABA in driving the gamma response in humans (Muthukumaraswamy, 2014), our observations may result from neurochemical as well as structural changes in cortical networks, in typical Alzheimer’s disease and PCA.

While PCA and typical Alzheimer’s disease were both associated with similar changes in spectral density, they had different scalp distributions of abnormality that reflected the known distribution of underlying pathology, with a parietal peak for PCA and predominantly temporal distribution for typical Alzheimer’s disease, in keeping with our principal hypothesis. The regional distribution of abnormal MEG-based connectivity cannot be equated with MRI or PET findings. For example, our planar gradiometers will be relatively insensitive to bilateral precuneus pathology in Alzheimer’s disease by virtue of depth and orientation. The abnormal local efficiency in typical Alzheimer’s disease also appears to be more extensive on the left, as has been noted before (Long *et al.*, 2013), but such laterality effects should be viewed with caution, without inferring unilateral pathology: indeed by the stage of clinically diagnosed, symptomatic Alzheimer’s disease, both amyloid and tau pathologies are likely to be bilateral and widespread (Jagust, 2016; Ossenkoppele *et al.*, 2016; Passamonti *et al.*, 2017). If spatio-temporal connectivity signatures are to provide a framework to understand multiple neurodegenerative disorders, complementary signatures are predicted for other pathologies and syndromes, to which we turn in the next section.

### Frontotemporal lobar degeneration syndromes

We studied three syndromes associated with FTLD: bvFTD, navPPA and classical PSP (Richardson’s syndrome). The clinicopathological correlation of PSP is very high, with 90–95% of cases due to a glioneuronal 4-repeat tauopathy (Litvan *et al.*, 1996; Frank *et al.*, 2007). Eleven of our PSP patients have since died, of whom seven donated their brain to the Cambridge Brain Bank: all seven had pathological confirmation of PSP. MRI studies reveal PSP-related atrophy of medial frontal cortex (Ghosh *et al.*, 2012) and the loss of frontocentral local efficiency in Fig. 2 accords with this structural change. The MEG analysis revealed a selective loss in low frequency connectivity up to the high beta range.

NavPPA has weaker clinicopathological correlations, but it is also associated with tauopathy in the majority of cases, while a minority have TDP-43 pathology (Gorno-Tempini *et al.*, 2011). Atrophy is typically not severe. BvFTD is the most neuropathologically diverse form, and may arise from 3-repeat or 4-repeat tauopathy or TDP-43 pathology (Boeve, 2007). The clinical syndrome is united by specific and severe layer II/III atrophy of temporal poles, plus moderate to severe atrophy of orbital and ventral frontal cortex (Hughes *et al.*, 2015). Like PSP, both navPPA and bvFTD reduced the local efficiency in the delta and beta bands, but the spatial and temporal characteristics of these changes were specific, with near complete separation of bvFTD and navPPA from the other syndromes (Fig. 3) reinforcing the multimodal separation of Alzheimer’s disease from navPPA (Hu *et al.*, 2010).

During our classification procedure, bvFTD was not well separated from PSP, which is interesting in view of the phenotypic overlap, given that PSP can present cognitive and behavioural change and many patients with bvFTD later develop a supranuclear gaze palsy and/or parkinsonism (Burrell *et al.*, 2014; Coyle-Gilchrist *et al.*, 2016; Höglinger *et al.* 2017). Here, both PSP and bvFTD were associated with loss of low frequency connectivity, in keeping with animal models of tau-mediated FTLD. For example, PLB2-tau mice show absolute power reductions in alpha band (9–14 Hz) in frontal and parietal locations (Koss *et al.*, 2016).

### Network-based biomarkers of neurodegeneration

The connectivity approach is ideally suited to the distributed nature of neuropathology and the impact of disease on the axon and synapse. Covariance-based resting state networks identified from MEG/EEG are reliable and sensitive to a wide range of neurodegenerative diseases (Stam, 2010; Hughes and Rowe, 2013). However, our use of directed graphs, or effective connectivity embodying directionality, extends this work and accommodates potential asymmetries in large-scale brain networks. MEG and EEG allow one to identify reciprocal connections across a range of frequencies: this makes them well suited to characterize the impact of dementia on connectivity *in vivo*, while maintaining compatibility with invasive electrophysiological studies of networks (Muthukumaraswamy, 2014; Phillips *et al.*, 2015).

We used PDC to quantify connectivity, a method related to Granger causality. PDC estimates directional connectivity between regions based on their functional time series. An advantage of this method for MEG/EEG is that it is less sensitive to the field spread that otherwise inflates instantaneous correlation metrics (Baccalá and Sameshima, 2001; van Dellen *et al.*, 2013; Colclough *et al.*, 2016). When combined with the focal field-of-view of planar gradiometers, simulation studies confirm that multivariate autoregressive modelling minimizes field spread while remaining veridical to source–space interactions (Pereira *et al.*, 2017). Indeed, our own simulations confirmed that this approach can recover average local efficiency of source-level networks, provided signal-to-noise ratio is sufficiently high (Supplementary Fig. 2). The use of sensor-level PDC avoids the extra assumptions that are needed to optimize the electromagnetic ‘inverse problem’ (Baillet *et al.*, 2001). However, we acknowledge that planar gradiometers are only sensitive to relative superficial cortical activity, and we may have missed the effects of disease in deeper brain structures (such information might be present in magnetometer data, but would require source reconstruction to infer network properties).

PDC informed the graph theoretical measures of network function. Graph theory reveals fundamental properties of brain network organization in health and has shown homologous vulnerabilities across many neurological and psychiatric disorders (Stam, 2014). The network-level description supports comparisons across modalities, scales and disorders (Fornito *et al.*, 2015). There are many measures of global network properties, such as small-worldness or global efficiency, but we focus here on local efficiency for two reasons. First, many neurodegenerative diseases are characterized by regional rather than global pathology. Second, it describes the local information transfer and resilience of a network. Third, previous studies have suggested that local efficiency, and its counterpart of local clustering, are impaired by neurodegeneration and can be sensitive to pathology even in the absence of focal atrophy (Stam, 2014). Recent neuroimaging-based network models have identified local network efficiency as one of the strongest indicators of underlying pathology in Alzheimer’s disease and frontotemporal dementia (Medaglia *et al.*, 2017). These properties make it ideal to elucidate the mechanisms of lobar neurodegenerative disorders with differing atrophic burdens (Seeley *et al.*, 2009; de Haan *et al.*, 2012*a*; van Dellen *et al.*, 2013; Stam, 2014; Hughes *et al.*, 2015). It should be noted that our measures of local efficiency are derived from directed, but binarized, connections. The direction of connections is important because it affects the local efficiency measure. It is possible that weighted (rather than binarized) connections would further inform graph metrics, but one cannot compare PDC values across different sending sensors, so binarization is required (which we implemented here by thresholding the highest 85% of PDC values).

There are limitations to our study. Severe atrophy is characteristic of typical Alzheimer’s disease, PCA and bvFTD (Rabinovici *et al.*, 2007; Crutch *et al.*, 2012; Whitwell and Josephs, 2012), and this might influence the sensors’ sensitivity to cortical sources and their connectivity. However, focal cortical atrophy in PSP and navPPA is usually mild or absent (Cope *et al.*, 2018), even though it can be evident in group studies (Ghosh *et al.*, 2012; Mandelli *et al.*, 2016). Moreover, a simple loss of sensitivity due to atrophy would not be a sufficient explanation of our results. The selective impairment of certain frequency bands suggests that our results are not merely a result of volume loss and increased distance from source to sensor: this is likely to affect all frequencies and be less reduced in PSP and navPPA (Bastos and Schoffelen, 2016). The frequency-specificity of group differences also argue against a simple model of cortical oscillatory dynamics in which higher frequencies are nested in low frequency oscillations (Lakatos *et al.*, 2008; Lisman and Jensen, 2013). This may be due to the selective impact of Alzheimer’s disease and FTLD on superficial and deep cortical layers, or to the selective breakdown of the neurochemical modulation of brain states (Uhlhaas and Singer, 2006; Murley and Rowe, 2018).

Our analyses focus on sensor space using planar gradiometers, rather than magnetometres or attempting to reconstruct source space activity. Several methods exist that reconstruct source space activity. However, the accuracy of these methods in conjunction with graphical network analysis is not yet established, and the good approximation of planar gradiometer topography to underlying cortical sources provides sufficient resolution to test our current hypotheses. Simulation studies confirm that multivariate auto-regression modelling is more robust in sensor space (Michalareas *et al.*, 2013), while the use of lagged interaction measures from planar gradiometer data are less sensitive to field-spread (Pereira *et al.*, 2017). Our own simulations provided further evidence that the analysis of sensor space graph metrics accords with source space generators of the data.

Another limitation is that our groups are defined by clinical diagnostic criteria, without pathological or genetic confirmation except for PSP. However, all our patient participants had well established disease, not peri-symptomatic or mild cognitive impairment. With this degree of severity, the clinicopathological correlations are high for PSP, typical Alzheimer’s disease, and PCA. Pathology would be of interest in bvFTD to differentiate those with tau versus TDP43 pathology, although consensus clinical diagnostic criteria are reliable in separating bvFTD from Alzheimer’s disease. There are potentially significant effects of age on MEG-derived power spectra (Tsvetanov *et al.*, 2015). These might confound the PCA results, being younger than other groups, although such age effects would not explain the spectral ‘similarity’ between PCA and typical Alzheimer’s disease, or the differences between typical Alzheimer’s disease and other groups.

In conclusion, the local efficiency of cortical networks was impaired by each of five neurodegenerative syndromes resulting from Alzheimer’s disease and FTLD. The five disorders had distinctive profiles in terms of their distribution and frequency of oscillatory activity. Pattern classification using the spatiotemporal map of connectivity differentiated the five disorders. The frequency range of the loss of local efficiency was distinguished by the likely underlying pathology, while the anatomical distribution related to the clinical syndrome. These findings enrich preclinical models of the physiological consequence of neuropathology, and link clinical *in vivo* measures to preclinical models of degeneration. They also provide potential physiological biomarkers with which to assess pre-symptomatic network dysfunction in early stage disease and for tracking disease progression or disease-modifying therapies in experimental medicine studies.

## Funding

This work is funded by the Wellcome Trust (103838) with additional support from the Medical Research Council (MC-A060‐5PQ30, MC-A060‐0046 and RG62761), Alzheimer’s Research UK, the NIHR Cambridge Biomedical Research Centre including the Cambridge Brain Bank, the Association of British Neurologists (T.E.C.) and the James S. McDonnell Foundation Understanding Human Cognition Scholar Award (J.B.R.)

## Supplementary material

Supplementary material is available at *Brain* online.

## Supplementary Material

Supplementary Data

## Abbreviations

bvFTD: behavioural variant frontotemporal dementia
FTLD: frontotemporal lobar degeneration
MEG: magnetoencephalography
MVAR: multivariate autoregressive modelling
navPPA: non-fluent agrammatic variant of primary progressive aphasia
PCA: posterior cortical atrophy
PDC: partial directed coherence
PSP: progressive supranuclear palsy

## References

[awy180-B1] Baccalá LA, Sameshima K Partial directed coherence: a new concept in neural structure determination. Biol Cybern 2001; 84: 463–74. 11417058 10.1007/PL00007990

[awy180-B2] Baillet S, Riera JJ, Marin G, Mangin JF, Aubert J, Garnero L Evaluation of inverse methods and head models for EEG source localization using a human skull phantom. Phys Med Biol 2001; 46: 77–96. 11197680 10.1088/0031-9155/46/1/306

[awy180-B3] Bastos AM, Schoffelen J-M A tutorial review of functional connectivity analysis methods and their interpretational pitfalls. Front Syst Neurosci 2016; 9: 175. 26778976 10.3389/fnsys.2015.00175 PMC4705224

[awy180-B4] Boeve BF Links between frontotemporal lobar degeneration, corticobasal degeneration, progressive supranuclear palsy, and amyotrophic lateral sclerosis: Alzheimer Dis Assoc Disord 2007; 21: S31–8. 18090421 10.1097/WAD.0b013e31815bf454

[awy180-B5] Bullmore E, Sporns O Complex brain networks: graph theoretical analysis of structural and functional systems. Nat Rev Neurosci 2009; 10: 186–98. 19190637 10.1038/nrn2575

[awy180-B6] Burrell JR, Hodges JR, Rowe JB Cognition in corticobasal syndrome and progressive supranuclear palsy: a review. Mov Disord 2014; 29: 684–93. 24757116 10.1002/mds.25872

[awy180-B7] Chan D, Walters RJ, Sampson EL, Schott JM, Smith SJ, Rossor MN EEG abnormalities in frontotemporal lobar degeneration. Neurology 2004; 62: 1628–30. 15136699 10.1212/01.wnl.0000123103.89419.b7

[awy180-B8] Colclough GL, Woolrich MW, Tewarie PK, Brookes MJ, Quinn AJ, Smith SM How reliable are MEG resting-state connectivity metrics? Neuroimage 2016; 138: 284–93. 27262239 10.1016/j.neuroimage.2016.05.070 PMC5056955

[awy180-B9] Coyle-Gilchrist ITS, Dick KM, Patterson K, Rodríquez PV, Wehmann E, Wilcox A et al Prevalence, characteristics, and survival of frontotemporal lobar degeneration syndromes. Neurology 2016; 86: 1736–43. 27037234 10.1212/WNL.0000000000002638 PMC4854589

[awy180-B10] Cope TE, Rittman T, Borchert RJ, Jones PS, Vatansever D, Allinson K et al Tau burden and the functional connectome in Alzheimer’s disease and progressive supranuclear palsy. Brain 2018; 141: 550–67. 29293892 10.1093/brain/awx347 PMC5837359

[awy180-B11] Crossley NA, Mechelli A, Scott J, Carletti F, Fox PT, McGuire P et al The hubs of the human connectome are generally implicated in the anatomy of brain disorders. Brain 2014; 137: 2382–95. 25057133 10.1093/brain/awu132 PMC4107735

[awy180-B12] Crutch SJ, Lehmann M, Schott JM, Rabinovici GD, Rossor MN, Fox NC Posterior cortical atrophy. Lancet Neurol 2012; 11: 170–8. 22265212 10.1016/S1474-4422(11)70289-7 PMC3740271

[awy180-B13] Dauwels J, Vialatte F, Musha T, Cichocki A A comparative study of synchrony measures for the early diagnosis of Alzheimer’s disease based on EEG. Neuroimage 2010; 49: 668–93. 19573607 10.1016/j.neuroimage.2009.06.056

[awy180-B14] Fornito A, Zalesky A, Breakspear M The connectomics of brain disorders. Nat Rev Neurosci 2015; 16: 159–72. 25697159 10.1038/nrn3901

[awy180-B15] Frank S, Clavaguera F, Tolnay M Tauopathy models and human neuropathology: similarities and differences. Acta Neuropathol 2007; 115: 39–53. 17786456 10.1007/s00401-007-0291-9

[awy180-B16] Ghosh BCP, Calder AJ, Peers PV, Lawrence AD, Acosta-Cabronero J, Pereira JM et al Social cognitive deficits and their neural correlates in progressive supranuclear palsy. Brain 2012; 135: 2089–102. 22637582 10.1093/brain/aws128 PMC3381722

[awy180-B17] Gonzalez-Moreno A, Aurtenetxe S, Lopez-Garcia M-E, del Pozo F, Maestu F, Nevado A Signal-to-noise ratio of the MEG signal after preprocessing. J Neurosci Methods 2014; 222: 56–61. 24200506 10.1016/j.jneumeth.2013.10.019

[awy180-B18] Gorno-Tempini ML, Hillis AE, Weintraub S, Kertesz A, Mendez M, Cappa SF et al Classification of primary progressive aphasia and its variants. Neurology 2011; 76: 1006–14. 21325651 10.1212/WNL.0b013e31821103e6 PMC3059138

[awy180-B19] de Haan W, Mott K, van Straaten ECW, Scheltens P, Stam CJ Activity dependent degeneration explains hub vulnerability in Alzheimer’s disease. PLoS Comput Biol2012a; 8: e1002582. 22915996 10.1371/journal.pcbi.1002582 PMC3420961

[awy180-B20] de Haan W, van der Flier WM, Koene T, Smits LL, Scheltens P, Stam CJ Disrupted modular brain dynamics reflect cognitive dysfunction in Alzheimer’s disease. Neuroimage2012b; 59: 3085–93. 22154957 10.1016/j.neuroimage.2011.11.055

[awy180-B22] Hayasaka S, Nichols TE Combining voxel intensity and cluster extent with permutation test framework. Neuroimage 2004; 23: 54–63. 15325352 10.1016/j.neuroimage.2004.04.035

[awy180-B23] Hillman EMC Coupling mechanism and significance of the BOLD signal: a status report. Annu Rev Neurosci 2014; 37: 161–81. 25032494 10.1146/annurev-neuro-071013-014111 PMC4147398

[awy180-B24] Höglinger GU, Respondek G, Stamelou M, Kurz C, Josephs KA, Lang AE et al Clinical diagnosis of progressive supranuclear palsy: the movement disorder society criteria. Mov Disord 2017; 32: 853–64. 28467028 10.1002/mds.26987 PMC5516529

[awy180-B25] Hu WT, McMillan C, Libon D, Leight S, Forman M, Lee VM et al Multimodal predictors for Alzheimer disease in nonfluent primary progressive aphasia. Neurology 2010; 75: 595–602. 20713948 10.1212/WNL.0b013e3181ed9c52 PMC2931765

[awy180-B26] Huey ED, Putnam KT, Grafman J A systematic review of neurotransmitter deficits and treatments in frontotemporal dementia. Neurology 2006; 66: 17–22. 16401839 10.1212/01.wnl.0000191304.55196.4d PMC4499854

[awy180-B27] Hughes LE, Ghosh BCP, Rowe JB Reorganisation of brain networks in frontotemporal dementia and progressive supranuclear palsy. Neuroimage Clin 2013; 2: 459–68. 23853762 10.1016/j.nicl.2013.03.009 PMC3708296

[awy180-B28] Hughes LE, Rittman T, Regenthal R, Robbins TW, Rowe JB Improving response inhibition systems in frontotemporal dementia with citalopram. Brain 2015; 138: 1961–75. 26001387 10.1093/brain/awv133 PMC5412666

[awy180-B29] Hughes LE, Rowe JB The impact of neurodegeneration on network connectivity: a study of change detection in frontotemporal dementia. J Cogn Neurosci 2013; 25: 802–13. 23469882 10.1162/jocn_a_00356 PMC3708294

[awy180-B30] Jagust W Is amyloid- β harmful to the brain? Insights from human imaging studies. Brain 2016; 139: 23–30. 26614753 10.1093/brain/awv326 PMC4990654

[awy180-B31] Knight RA, Verkhratsky A Neurodegenerative diseases: failures in brain connectivity? Cell Death Differ 2010; 17: 1069–70. 20543854 10.1038/cdd.2010.23

[awy180-B32] Koss DJ, Robinson L, Drever BD, Plucińska K, Stoppelkamp S, Veselcic P et al Mutant Tau knock-in mice display frontotemporal dementia relevant behaviour and histopathology. Neurobiol Dis 2016; 91: 105–23. 26949217 10.1016/j.nbd.2016.03.002

[awy180-B33] Kurudenkandy FR, Zilberter M, Biverstål H, Presto J, Honcharenko D, Strömberg R et al Amyloid-β-induced action potential desynchronization and degradation of hippocampal gamma oscillations is prevented by interference with peptide conformation change and aggregation. J Neurosci 2014; 34: 11416–25. 25143621 10.1523/JNEUROSCI.1195-14.2014 PMC6615507

[awy180-B34] Lakatos P, Karmos G, Mehta AD, Ulbert I, Schroeder CE Entrainment of neuronal oscillations as a mechanism of attentional selection. Science 2008; 320: 110–13. 18388295 10.1126/science.1154735

[awy180-B35] Latora V, Marchiori M Efficient behavior of small-world networks. Phys Rev Lett 2001; 87: 198701. 11690461 10.1103/PhysRevLett.87.198701

[awy180-B37] Lisman JE, Jensen O The theta-gamma neural code. Neuron 2013; 77: 1002–16. 23522038 10.1016/j.neuron.2013.03.007 PMC3648857

[awy180-B38] Litvan I, Agid Y, Calne D, Campbell G, Dubois B, Duvoisin RC et al Clinical research criteria for the diagnosis of progressive supranuclear palsy (Steele-Richardson-Olszewski syndrome): report of the NINDS-SPSP international workshop. Neurology 1996; 47: 1–9. 8710059 10.1212/wnl.47.1.1

[awy180-B39] Long X, Zhang L, Liao W, Jiang C, Qiu B Distinct laterality alterations distinguish mild cognitive impairment and Alzheimer’s disease from healthy aging: statistical parametric mapping with high resolution MRI. Hum Brain Mapp 2013; 34: 3400–10. 22927141 10.1002/hbm.22157 PMC6870259

[awy180-B40] Maestu F, Pena JM, Garces P, Gonzalez S, Bajo R, Bagic A Magnetoencephalography international consortium of Alzheimer’s, D. A multicenter study of the early detection of synaptic dysfunction in mild cognitive impairment using magnetoencephalography-derived functional connectivity. Neuroimage Clin 2015; 9: 103–9. 26448910 10.1016/j.nicl.2015.07.011 PMC4552812

[awy180-B42] Mandelli ML, Vilaplana E, Brown JA, Hubbard HI, Binney RJ, Attygalle S et al Healthy brain connectivity predicts atrophy progression in non-fluent variant of primary progressive aphasia. Brain 2016; 139: 2778–91. 27497488 10.1093/brain/aww195 PMC5035819

[awy180-B41] Medaglia JD, Huang W, Segarra S, Olm C, Gee J, Grossman M et al Brain network efficiency is influenced by the pathologic source of corticobasal syndrome. Neurology 2017; 89: 1373–81. 28779011 10.1212/WNL.0000000000004324 PMC5649755

[awy180-B43] Meier J, Zhou X, Hillebrand A, Tewarie P, Stam CJ, Van Mieghem P The epidemic spreading model and the direction of information flow in brain networks. Neuroimage 2017; 152: 639–46. 28179163 10.1016/j.neuroimage.2017.02.007

[awy180-B501] Michalareas G, Schoffelen JM, Paterson G, Gross J Investigating causality between interacting brain areas with multivariate autoregressive models of MEG sensor data. Hum Brain Mapp 2013; 34: 890–913. 22328419 10.1002/hbm.21482 PMC3617463

[awy180-B44] Moon JY, Lee U, Blain-Moraes S, Mashour GA General relationship of global topology, local dynamics, and directionality in large-scale brain networks. PLoS Comput Biol 2015; 11: e1004225. 25874700 10.1371/journal.pcbi.1004225 PMC4397097

[awy180-B45] McKhann GM, Knopman DS, Chertkow H, Hyman BT, Jack CR, Kawas CH et al The diagnosis of dementia due to Alzheimer’s disease: recommendations from the National Institute on Aging-Alzheimer’s Association workgroups on diagnostic guidelines for Alzheimer’s disease. Alzheimers Dement J Alzheimers Assoc 2011; 7: 263–9. 10.1016/j.jalz.2011.03.005 PMC3312024 21514250

[awy180-B502] Murley AG, Rowe JB Neurotransmitter deficits from frontotemporal lobar degeneration. Brain 2018; 141: 1263–85. 29373632 10.1093/brain/awx327 PMC5917782

[awy180-B46] Muthukumaraswamy SD The use of magnetoencephalography in the study of psychopharmacology (pharmaco-MEG). J Psychopharmacol 2014: 28: 815–29. 24920134 10.1177/0269881114536790

[awy180-B47] Ossenkoppele R, Schonhaut DR, Schöll M, Lockhart SN, Ayakta N, Baker SL et al Tau PET patterns mirror clinical and neuroanatomical variability in Alzheimer’s disease. Brain 2016; 139: 1551–67. 26962052 10.1093/brain/aww027 PMC5006248

[awy180-B48] Passamonti L, Vazquez Rodriguez P, Hong YT, Allinson KS, Williamson D, Borchert RJ et al 18F-AV-1451 positron emission tomography in Alzheimer’s disease and progressive supranuclear palsy. Brain 2017; 140: 781–91. 28122879 10.1093/brain/aww340 PMC5382948

[awy180-B49] Palop JJ, Mucke L Amyloid-β-induced neuronal dysfunction in Alzheimer’s disease: from synapses toward neural networks. Nat Neurosci 2010; 13: 812–18. 20581818 10.1038/nn.2583 PMC3072750

[awy180-B50] Pena-Ortega F, Solis-Cisneros A, Ordaz B, Balleza-Tapia H, Javier Lopez-Guerrero J Amyloid beta 1‐42 inhibits entorhinal cortex activity in the beta-gamma range: role of GSK-3. Curr Alzheimer Res 2012; 9: 857–63. 22631612 10.2174/156720512802455403

[awy180-B51] Pereira S, Hindriks R, Miihlberg S, Maris E, van Ede F, Griffa A et al Effect of field spread on resting-state MEG functional network analysis: a computational modeling study. Brain Connectivity 2017; 7: 541–57. 28875718 10.1089/brain.2017.0525

[awy180-B52] Petersen RC, Doody R, Kurz A, Mohs RC, Morris JC, Rabins PV et al Current concepts in mild cognitive impairment. Arch Neurol 2001; 58: 1985–92. 11735772 10.1001/archneur.58.12.1985

[awy180-B53] Pievani M, de Haan W, Wu T, Seeley WW, Frisoni GB Functional network disruption in the degenerative dementias. Lancet Neurol 2011: 10: 829–43. 21778116 10.1016/S1474-4422(11)70158-2 PMC3219874

[awy180-B54] Phillips HN, Blenkmann A, Hughes LE, Bekinschtein TA, Rowe JB Hierarchical organization of frontotemporal networks for the prediction of stimuli across multiple dimensions. J Neurosci 2015; 35: 9255–64. 26109651 10.1523/JNEUROSCI.5095-14.2015 PMC4478247

[awy180-B55] Poil SS, de Haan W, van der Flier WM, Mansvelder HD, Scheltens P, Linkenkaer-Hansen K Integrative EEG biomarkers predict progression to Alzheimer’s disease at the MCI stage. Front Aging Neurosci 2013; 5: 58. 24106478 10.3389/fnagi.2013.00058 PMC3789214

[awy180-B56] Poza J, Hornero R, Abasolo D, Fernandez A, Escudero J Analysis of spontaneous MEG activity in patients with Alzheimer’s disease using spectral entropies. In: 2007 29th Annual international conference of the IEEE engineering in medicine and biology society. IEEE EMBS, 2007. p. 6179–82. doi: 10.1109/IEMBS.2007.4353766. 10.1109/IEMBS.2007.4353766 18003432

[awy180-B57] Quiroz YT, Ally BA, Celone K, McKeever J, Ruiz-Rizzo AL, Lopera F et al Event-Related potential markers of brain changes in preclinical familial Alzheimer disease. Neurology 2011: 77: 469–75. 21775732 10.1212/WNL.0b013e318227b1b0 PMC3146305

[awy180-B58] Rabinovici GD, Seeley WW, Kim EJ, Gorno-Tempini ML, Rascovsky K, Pagliaro TA et al Distinct MRI atrophy patterns in autopsy-proven Alzheimer’s disease and frontotemporal lobar degeneration. Am J Alzheimers Dis Other Demen 2007; 22: 474–88. 18166607 10.1177/1533317507308779 PMC2443731

[awy180-B59] Raj A, Kuceyeski A, Weiner M A network diffusion model of disease progression in dementia. Neuron 2012; 73: 1204–15. 22445347 10.1016/j.neuron.2011.12.040 PMC3623298

[awy180-B60] Rascovsky K, Hodges JR, Knopman D, Mendez MF, Kramer JH, Neuhaus J et al Sensitivity of revised diagnostic criteria for the behavioural variant of frontotemporal dementia. Brain 2011; 134: 2456–77. 21810890 10.1093/brain/awr179 PMC3170532

[awy180-B61] Seeley WW, Crawford RK, Zhou J, Miller BL, Greicius MD Neurodegenerative diseases target large-scale human brain networks. Neuron 2009; 62: 42–52. 19376066 10.1016/j.neuron.2009.03.024 PMC2691647

[awy180-B62] Selkoe DJ Alzheimer’s disease is a synaptic failure. Science 2002; 298: 789–91. 12399581 10.1126/science.1074069

[awy180-B160] Schelter B, Winterhalder M, Eichler M, Peifer M, Hellwig B, Guschlbauer B et al Testing for directed influences among neural signals using partial directed coherence. J Neurosci Methods 2006; 152: 210–9. 16269188 10.1016/j.jneumeth.2005.09.001

[awy180-B63] Spires-Jones TL, Hyman BT The intersection of amyloid beta and tau at synapses in Alzheimer’s disease. Neuron 2014; 82: 756–71. 24853936 10.1016/j.neuron.2014.05.004 PMC4135182

[awy180-B64] Sporns O, Honey CJ, Kötter R Identification and classification of hubs in brain networks. PLoS One 2007; 2: e1049. 17940613 10.1371/journal.pone.0001049 PMC2013941

[awy180-B65] Stam CJ Use of magnetoencephalography (MEG) to study functional brain networks in neurodegenerative disorders. J Neurol Sci 2010; 289: 128–34. 19729174 10.1016/j.jns.2009.08.028

[awy180-B66] Stam CJ Modern network science of neurological disorders. Nat Rev Neurosci 2014; 15: 683–95. 25186238 10.1038/nrn3801

[awy180-B67] Triggiani AI, Bevilacqua V, Brunetti A, Lizio R, Tattoli G, Cassano F et al Classification of healthy subjects and Alzheimer’s disease patients with dementia from cortical sources of resting state EEG rhythms: a study using artificial neural networks. Front Neurosci 2017; 10: 604. 28184183 10.3389/fnins.2016.00604 PMC5266711

[awy180-B68] Tsvetanov KA, Henson RN, Tyler LK, Davis SW, Shafto MA, Taylor JR et al The effect of ageing on fMRI: correction for the confounding effects of vascular reactivity evaluated by joint fMRI and MEG in 335 adults. Hum Brain Mapp 2015; 36: 2248–69. 25727740 10.1002/hbm.22768 PMC4730557

[awy180-B69] Uhlhaas PJ, Singer W Neural synchrony in brain disorders: relevance for cognitive dysfunctions and pathophysiology. Neuron 2006; 52: 155–68. 17015233 10.1016/j.neuron.2006.09.020

[awy180-B70] van Dellen E, Hillebrand A, Douw L, Heimans JJ, Reijneveld JC, Stam CJ Local polymorphic delta activity in cortical lesions causes global decreases in functional connectivity. Neuroimage 2013; 83: 524–32. 23769919 10.1016/j.neuroimage.2013.06.009

[awy180-B71] Whitwell JL, Josephs KA Recent advances in the imaging of frontotemporal dementia. Curr Neurol Neurosci Rep 2012; 12: 715–23. 23015371 10.1007/s11910-012-0317-0 PMC3492940

[awy180-B72] Williams N, Henson R, Taylor J Cam-CAN Measuring effective connectivity in resting-state MEG using PDC: effect of ageing in the Cam-CAN project. Poster presented at the Annual Scientific Meeting of the Organization for Human Brain Mapping (OHBM), Hamburg, Germany, 10 June 2014.

[awy180-B73] Yu M, Engels MMA, Hillebrand A, van Straaten ECW, Gouw AA, Teunissen C et al Selective impairment of hippocampus and posterior hub areas in Alzheimer’s disease: an MEG-based multiplex network study. Brain 2017; 140: 1466–85. 28334883 10.1093/brain/awx050

[awy180-B74] Zhou J, Greicius MD, Gennatas ED, Growdon ME, Jang JY, Rabinovici GD et al Divergent network connectivity changes in behavioural variant frontotemporal dementia and Alzheimer’s disease. Brain J Neurol 2010; 133: 1352–67. 10.1093/brain/awq075 PMC2912696 20410145

[awy180-B75] Zhou J, Gennatas ED, Kramer JH, Miller BL, Seeley WW Predicting regional neurodegeneration from the healthy brain functional connectome. Neuron 2012; 73: 1216–27. 22445348 10.1016/j.neuron.2012.03.004 PMC3361461

