# Supplementary material for: Neurophysiological signatures of Alzheimer’s disease and frontotemporal lobar degeneration: pathology versus phenotype
Source: Brain. 2018 Jul 9;141(8):2500–10. doi: 10.1093/brain/awy180 (PMC6061803; doi:10.1093/brain/awy180)
Supplement: Supplementary Data [file awy180_brain-2017-01746-file005.pdf]

Supplementary material

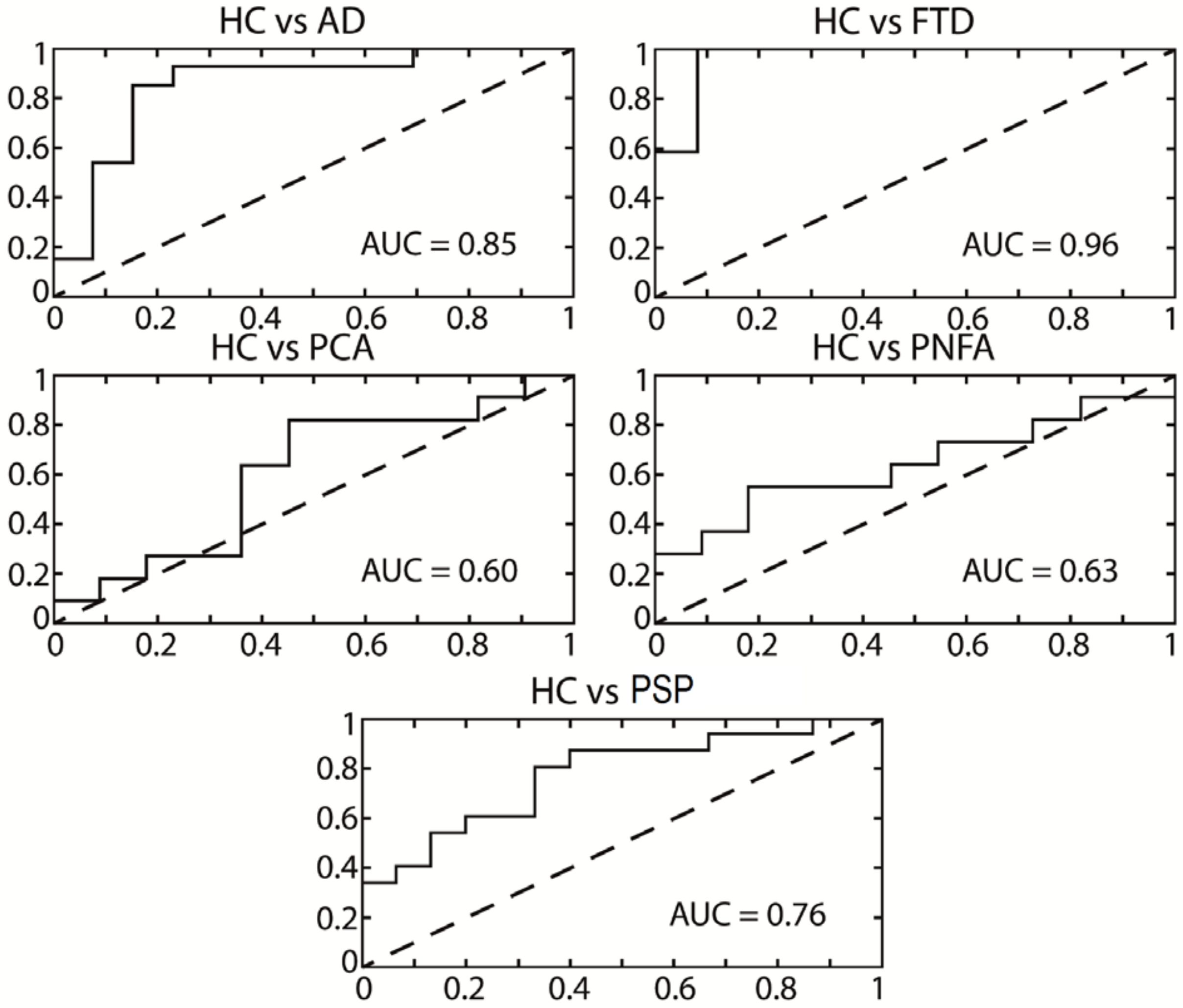

Supplementary Figure 1. Receiver operating characteristic (ROC) curves illustrate the performance of the Support vector machine classifier following principle component feature extraction under binary classifications between groups. The classification performance between Healthy Controls (HC) and patient groups is summarized by the area under the curve (AUC, inset). The y-axis represents the true positive rate while the x- axis represents the false positive rate. tAD = typical Alzheimer Disease; PCA = posterior cortical atrophy; bvFTD = behavioral variant frontotemporal dementia; navPPA= non-fluent variant primary progressive aphasia; PSP = progressive supranuclear palsy.

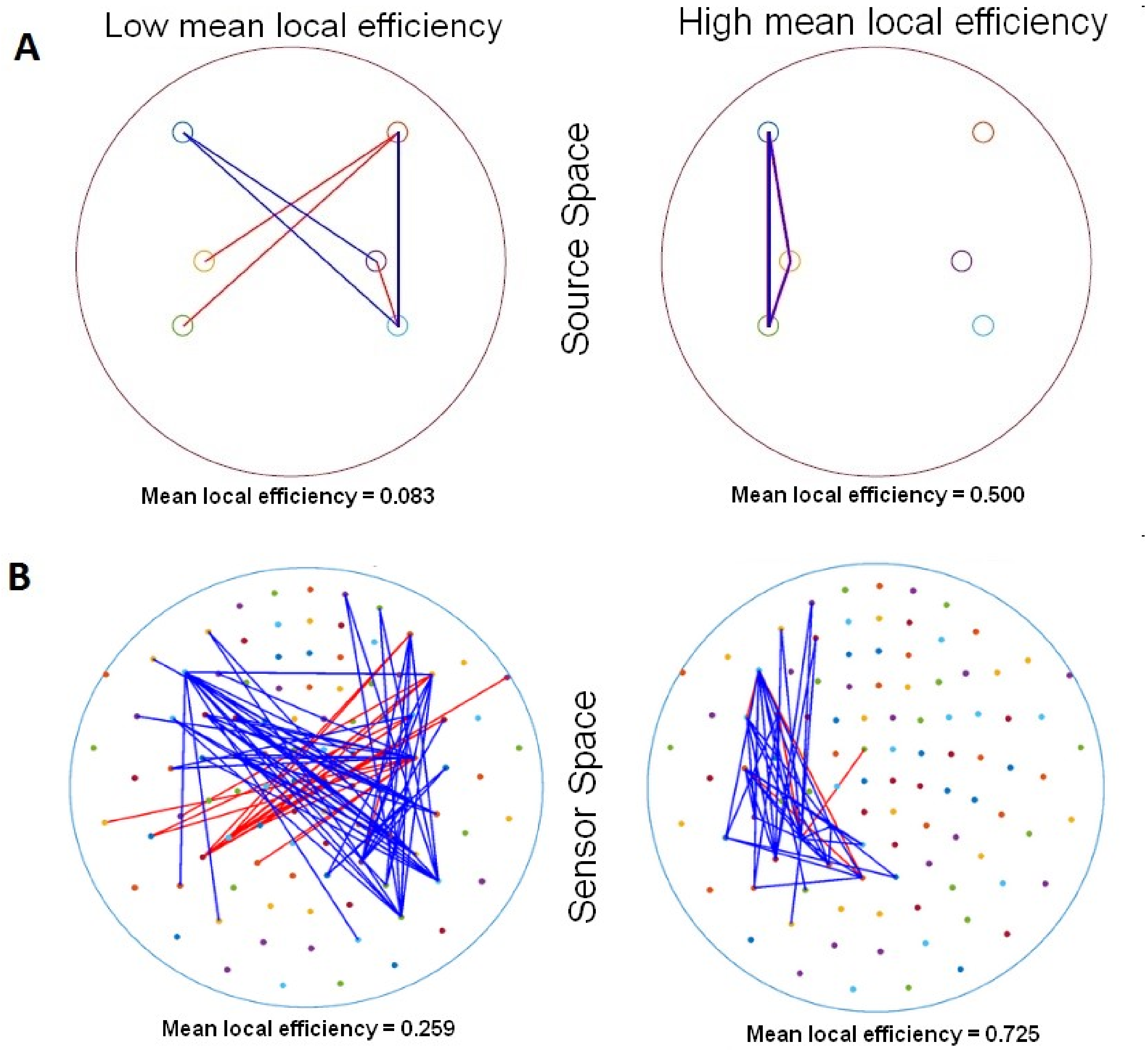

Supplementary Figure 2. shows one example each of a low and high efficiency network at source-level (A) and sensor- level (B) respectively, that were used in the simulations. The coarse topology of the source- level networks are preserved in the sensor-level networks. The difference in the mean local efficiency of the source-level networks is also preserved in the corresponding sensor-level networks, as in this example, using the 85% threshold and SNR=Inf (illustrated with the top 5% connections for visual clarity, with red vs blue lines differentiating the direction of connection between sources/sensors).

Supplementary Table 1

	85 percentile	95 percentile	99 percentile
SNR = 0.1	0.7933	0.8981	0.9805
1	0.7262	0.546	0.9132
3	0.0063	0.0025	0.1278
10	0.0049	4.5E-06	5.2E-07
Inf.	0.000024	4.6E-07	5.3E-06

Supplementary Table 1. shows the p values at five separate SNR intervals for three threshold levels. The table confirms that differences in mean local efficiency of the source-level networks were preserved in local efficiency of the corresponding sensor-level networks down to SNR=3, for thresholds of 85% and 95%, and down to SNR=10 for percentile threshold of 99%
